# [Retracted] miR-218 suppresses cardiac myxoma proliferation by targeting myocyte enhancer factor 2D

**DOI:** 10.3892/or.2026.9072

**Published:** 2026-02-09

**Authors:** Quanxing Cao, Pingshuan Dong, Yanyu Wang, Junwei Zhang, Xinge Shi, Yongsheng Wang

Oncol Rep 33: 2606–2612, 2015; DOI: 10.3892/or.2015.3861

Following the publication of the above paper, it was drawn to the Editor's attention by a concerned reader that the GAPDH and MEF21 protein bands shown in the western blots in Fig. 2C on p. 2608 were strikingly similar to data appearing in different form in other articles written by different authors at different research institutes that had either already been published, or were under consideration for publication at the same time, some of which have subsequently been retracted. Upon conducting an independent evaluation of the data in the Editorial Office, it emerged that the western blots shown in Fig. 1B in this paper had similarly appeared in a number of unrelated articles.

In view of the fact that the abovementioned data in Figs. 1B and 2C had already apparently been published previously, the Editor of *Oncology Reports* has decided that this paper should be retracted from the Journal. The authors were asked for an explanation to account for these concerns, but the Editorial Office did not receive a reply. The Editor apologizes to the readership for any inconvenience caused.

